# Impact of radiation dose on local control and survival in extramedullary head and neck plasmacytoma

**DOI:** 10.1186/s13014-019-1265-5

**Published:** 2019-04-15

**Authors:** Michael Oertel, Khaled Elsayad, Kai Jannes Kroeger, Uwe Haverkamp, Claudia Rudack, Georg Lenz, Hans Theodor Eich

**Affiliations:** 1 Department of Radiation Oncology, University Hospital of Muenster, Albert-Schweitzer-Campus 1, Building A1, 48149 Muenster, Germany; 2 Department of Otorhinolaryngology, Head and Neck Surgery, University Hospital of Muenster, Muenster, Germany; 3 Department of Internal Medicine-A (Hematology, Oncology, Hemostaseology and Pulmonology), University Hospital Muenster, Muenster, Germany

**Keywords:** Extramedullary, Plasmocytoma, Radiotherapy, IMRT, Local control, Prognosis

## Abstract

**Background:**

Patients with plasma-cell neoplasia usually suffer from systemic disease, although a minority (< 5%) may present with solitary involvement of bone or soft tissue (extramedullary plasmacytoma (EMP)). Radiotherapy (RT) is a state-of-the-art treatment for these tumors offering long term curation.

**Methods and materials:**

Between January 2005 and January 2017, twenty-seven patients underwent RT at our institution. The aim of this study was to analyse the effectiveness of various RT doses for different forms of EMP.

**Results:**

A total of 33 radiation courses were administered to 27 patients with a median age of 56 years. The median RT dose was 45 Gy (range: 12–55.8). The local control rate was 76% (93% for primary EMP vs. 61% for the secondary EMP lesions; *P* < 0.05). A complete response (CR) rate to local RT was achieved for 42% lesions (67% for primary EMP vs. 22% for the secondary EMP lesions; *P* < 0.01). The overall response rate (ORR) for the EMP lesions treated with high-dose regimens (> 45 Gy) versus low-dose regimens (≤ 45 Gy) was 87% versus 67%, respectively (*P* = 0.2). The median survival with high-dose RT group was significantly longer (*P* = 0.02). In subgroups analysis, primary EMP patients treated with high-dose RT had a non-significant higher ORR (100% vs. 80%, respectively; *P* = 0.3,) longer duration of LC (*P* = 0.3) with a longer survival (*P* = 0.05) than patients in low-dose group. No significant difference has been detected in secondary EMP patients treated with high-dose RT regarding ORR (60% vs. 62%, respectively; *P* = 1), and survival (*P* = 0.4).

**Conclusion:**

RT is an efficacious treatment modality in the treatment of EMP. A radiation dose ≤45 Gy confer a comparable CR rate to high-dose regimens and appears to be an effective treatment for controlling local EMP progression. Radiation dose-escalation may be beneficial for particular subgroups of patients.

## Background

Plasma cell neoplasms compromise multiple myeloma as well as osseous and extrasosseous plasmocytoma and are defined by the World Health Organisation as diseases with clonal proliferation of heavy chain class-switched mature B-cells with a characteristic secretion of a monoclonal immunglubolin (M-protein) [1]. Isolated accumulations of plasma cell outside the bone are called extramedullary plamocytoma (EMP) and are predominantly found in the upper aerodigestive tract [1–3]. Other organs may be prone to EMP spread such as skin, lymph nodes, brain, spine, thorax, liver, urogenital and gastrointestinal tract, mammary tissue or extremities [4–12]. Its diagnosis requires a single extramedullary mass of clonal plasma cell with normal bone marrow histology, absence of end organ damages (anemia, hypercalcaemia, renal impairment, osteolysis) and only minimal serum or urine level of monoclonal immunglobuline [1, 3]. Literature reviews show typical occurrence between the fourth and seventh decade of life with a male predominance [1, 2, 4, 13, 14]. Local symptoms in the head-and-neck region include epistaxis, facial swelling or facial pain painless mass and visual disturbances as well as sensory or motor cranial nerve impairments [15, 16].

Radiotherapy (RT) is the treatment of choice for EMP with high local control rates and long-term curation [3, 17]. As in other hematologic disease, defining the adequate RT dose has taken center stage in the delicate balance between sufficient tumor control and potential toxicity. Whereas dose-deescalation is currently being investigated for lymphoma and leukemia, there is no stringent evidence to suggest low-dose regimes for plasmocytoma [18, 19, 32–34].

In this study we aim to investigate the impact of different radiation dose regime on tumor control and to identify possible prognostic factors.

## Methods and materials

A total of 33 radiation courses were administered to 27 patients (9 females, 18 males) between January 2005 and January 2017 (Table 1). The median age was 56 years (range: 42–86). The median RT dose was 45 Gy (range: 12–55.8). There were fifteen (56%) patients with primary EMP and twelve (44%) with secondary manifestations in the head and neck region. The median time interval between the onset of multiple myeloma (MM) and secondary EMP presentation was 43 months (range: 0–109), while only 3 patients (11%) developed EMP and MM synchronously. All patients underwent head and neck CT scans. Further investigation using MRI or PET-CT was performed in fourteen patients (42%). The most common sites were nasal and paranasal sinuses (*N* = 10, 30%), pharyngeal (*N* = 6, 18%), and cervical soft tissues (*N* = 6, 18%). Fifteen lesions (45%) were treated with high RT-doses (> 45 Gy) with a median radiation dose of 50.4 Gy [commonly apllied in patients with nasal/paranasal (60%) and pharyngeal (83%) manifestations]. The other eighteen lesions (55%) were treated with lower RT-doses up to 45 Gy (median: 33 Gy) in in 12 patients and were commonly applied in patients with orbital (83%) and cervical (83%), and cutaneo-muscular (60%) manifestations. Fourteen patients (52%) received systemic therapies prior or after RT course (11/12 of patients with secondary EMP and 12/15 of patients with primary EMP, *P* < .001). 33% of patients in high-dose RT group received systemic treatment versus 75% of patients in low-dose group (*P* = .054). Detailed treatment characteristics are presented in Table 2. Patients with solitary bone lesion have been excluded.

**Table 1:** Patient characteristics (*N* = 27)

Characteristic	Value	Percentage/range
Mean age at EMP Dx, y	56 y	42–86
Gender ratio	18 M: 9 F	
Type of EMP		
Primary	15/27	56%
Secondary	12/27	44%
Involved lymph nodes		
Yes	7/27	26%
No	20/27	74%
Bone infiltration/erosion		
Yes	13/27	26%
No	20/27	74%
Immunohistochemical analysis		
Kappa light chain restriction	11/27	41%
Lambda light chain restriction	9/27	33%
unknown	7/27	26%
Serum Beta-2-microglobulin		
Elevated	5/27	18%
Normal	7/27	26%
Unknown	15/27	56%
Serum protein		
Elevated	3/27	11%
Normal	17/27	63%
Low	3/27	11%
Unknown	4/27	15%
Serum protein immunofixation		
Positive	14/27	52%
Negative	9/27	33%
Unknown	4/27	15%
Serum LDH		
Elevated	12/27	44%
Normal	11/27	41%
Unknown	4/27	15%
Serum calcium		
Elevated	1/27	4%
Normal	21/27	78%
Low	2/27	7%
Unknown	3/27	11%
Prior or adjuvant therapies		
HSCT	12/27	44%
TBI	3/27	11%
Systemic immunochemotherapy	14/27	52%

*F* female, *M* male, *Med*. median, *LDH* lactate dehydrogenase, *HSCT* Hematopoietic stem cell transplantation, *TBI* total body irradiation

**Table 2:** Treatment characteristics for 33 extramedullary plasmocytoma lesions

Characteristic	Value	Percentage/range
Treatment parameters		
Med. radiation dose (range), Gy	45	12–55.8
≤ 45 Gy	18/33	55%
> 45 Gy	15/33	45%
Med. fraction dose (range), Gy	2	1.8–4.0
Med. treatment time, d	35	4–52
Sites of RT		
Nasal/paranasal	10/33	30%
Pharynx	6/33	18%
Orbital/epidural	6/33	18%
Cervical	6/33	18%
Cutaneous/muscular	5/33	16%
Type of RT		
Postoperative	11/33	33%
Definitive	22/33	67%
RT technique		
IMRT	14/33	42%
CRT	19/33	58%
Bone erosion		
Yes	13/33	40%
No	14/33	42%
Unknown	16/33	18%
Local response		
Complete response	14/33	43%
Partial response	11/33	33%
Stable	1/33	3%
Progression	3/33	6%
Unknow	5/33	15%

*Med* median, *CRT* conventional radiotherapy, *CTX* chemotherapy, *RT* radiotherapy, *IMRT* intensity-modulated radiotherapy

### Statistical analysis

All statistical analyses were conducted with SPSS version 25.0 software (IBM, Armonk, NY). Differences were considered statistically significant at a *P*-value ≤0.05. OS was calculated from the first day of RT. Local control was calculated from the initiation of RT until the time of documented local relapse or death. Time-dependent event curves were calculated using the Kaplan-Meier method and were compared using the log-rank test.

### Definition of response

EMP response was assessed during RT, and at a 3-month follow-up appointment clinically and radiologically. Complete response (CR) was defined as complete clinical regression of irradiated lesion, while partial response (PR) represented any response less than complete that showed > 50% radiological response. Local progression was defined as > 25% clinical progress of the lesions.

## Results

**Fig. 1:**
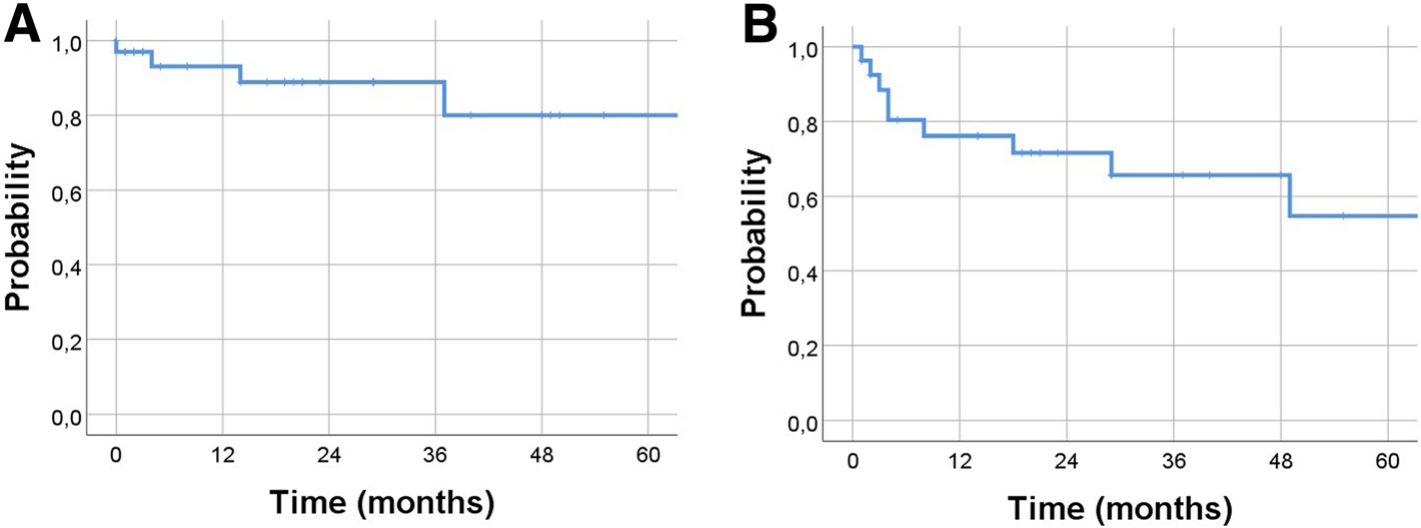
Kaplan–Meier estimates of local control (**a**) and overall survival for all patients (**b**)

The local control (LC) rate for the whole cohort was 76%. In subgroup analysis, the LC rate for primary EMP was 93% and for the secondary EMP lesions 61% (*P* < 0.05). CR after local RT was achieved for 42% lesions (67% for primary EMP vs. 22% for the secondary EMP lesions; *P* < 0.01). The 2-year LC rate of 89% and 5-year LC rate of 80% (Fig. 1**a**). In the whole cohort, the median OS has not been reached with 2-year OS of 72% and 5-year OS rate of 55% (Fig. 1**b**). The median duration of LC for the entire cohort has not been reached with no noticeable difference between primary and secondary EMP (*P* = .9). The 2-year LC rate in primary EMP patients was 92% versus 87% in secondary EMP (*P* = 0.9), respectively. The median OS from the time of RT was significantly longer in patients with primary EMP (*P* = .035). The 2-year OS rate was 85% versus 53%, respectively. Only two patients with primary EMP developed relapse outside the radiation fields (3 and 8 years after initial radiation course) and were treated successfully with salvage RT to cervical lymph node (36 Gy) and stomach (40 Gy), respectively.

**Fig. 2:**
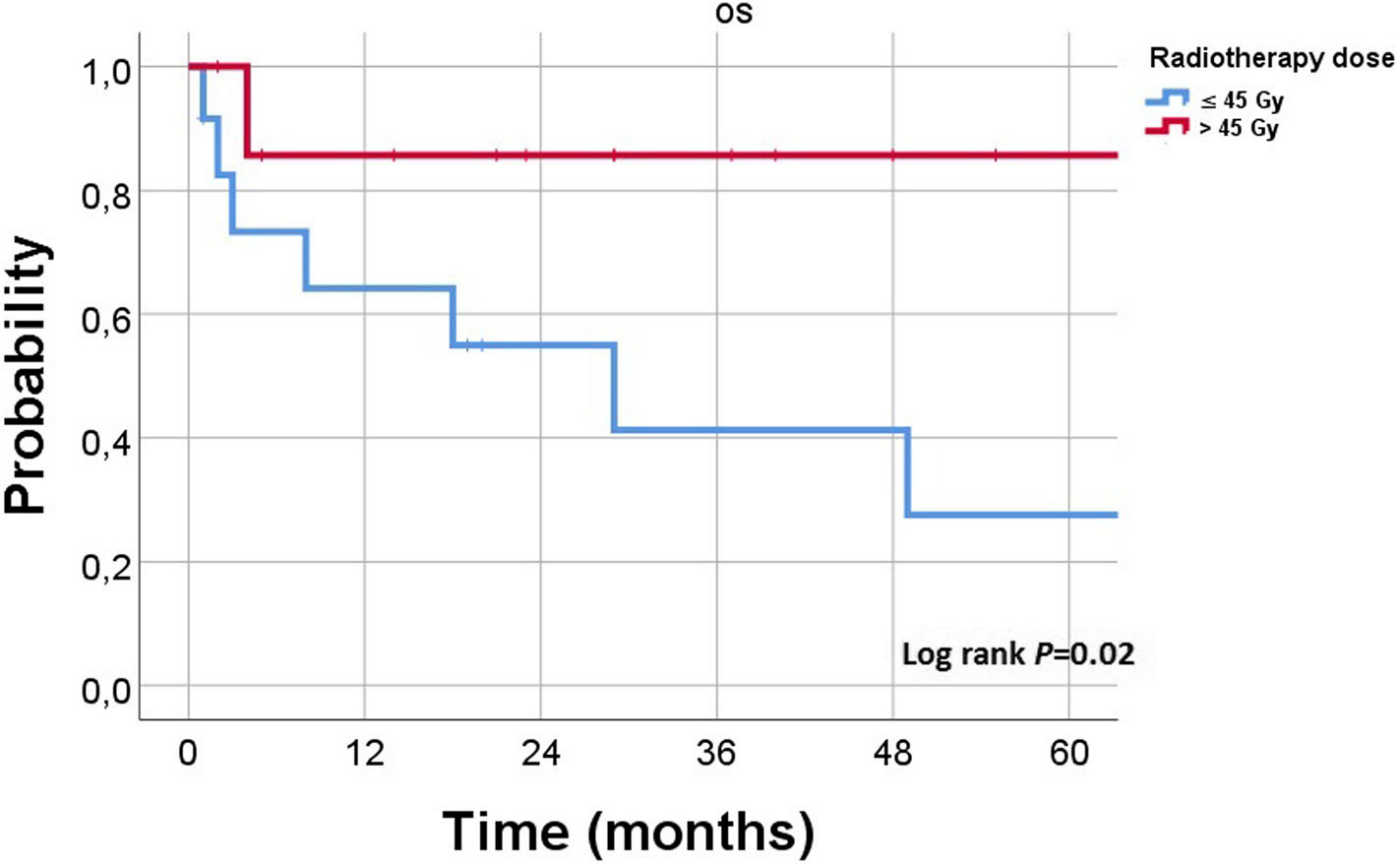
Kaplan–Meier estimates of overall survival according to radiation dose in all (*N* = 27) patients

**Fig. 3:**
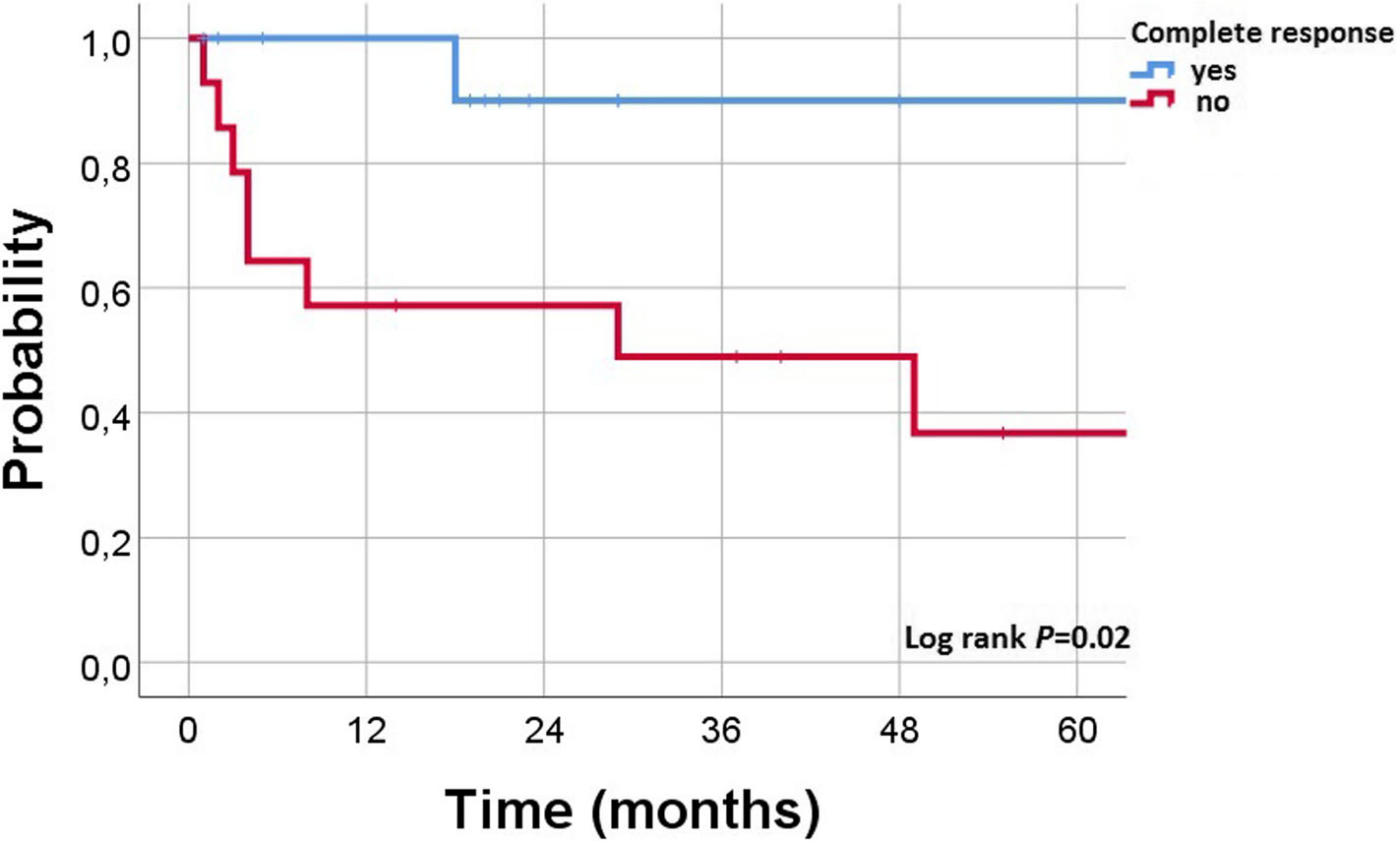
Kaplan–Meier estimates of overall survival for all patients and according to response after radiotherapy

Regarding the radiation dose, the overall response rate (ORR) for the EMP lesions treated with high-dose regimens versus low-dose regimens was 87% versus 67% (*P* = 0.2), and the CR rate was 53% versus 33%, respectively (*P* = 0.3). According to site of lesions, the ORR was higher following high-doses in nasal/paranasal (83% vs. 50%, *P* = .3), pharyngeal (100% in both therapy arms), orbital (100% vs. 60%, *P* = .7), and cervical (100% vs. 80%, *P* = .8) lesions. While in patients with cutaneo-muscular lesions the ORR was 50% following high-dose regimens versus 66% with low-doses (*P* = .8). In the whole cohort, the median survival with high-dose RT group was significantly longer (*P* = 0.02). The 2-year OS rate in the entire cohort was 86% with high-dose group versus 55% in low-dose group (Fig. 2, *P* = 0.02). In subgroups analysis, primary EMP patients treated with high-dose RT had a non-significant higher ORR (100% vs. 80%, respectively; *P* = 0.3) and longer duration of LC (*P* = 0.3) with a longer survival (*P* = 0.05) than patients in low-dose group. On the other hand, no significant difference has been detected in secondary EMP patients treated with high-dose RT regarding ORR (60% vs. 62%, respectively; *P* = 1), and survival (*P* = 0.4). However, DOLC was longer with low-dose group (*P* = 0.03). The 2-year OS of patients developed CR following RT was 90% with an platue till 5 years while other patients had 2-year OS of 57% and 5-year survival of 37% (Fig. 3, *P* = 0.02).

Regarding patients’ characteristics listed in Table 1, a non-significant improvement was seen in patients with normal β2-microglobulin (*P* = 0.1) and negative immunofixation (*P* = 0.16). While patients with normal lactat dehydrogenase (LDH value (*P* = 0.09) or negative immunofixation (*P* = 0.1) may impact the overall survival of EMP patients.

In the whole cohort, there was no LC advantage in patients who underwent surgical resection (*P* = 0.8). In contrast, better OS following postoperative RT in comparison with patients received RT alone has been observed (2-year OS of 89 versus 60%; *P* = 0.04), however this benefit couldn’t be seen in patients with primary EMP (*P* = 0.8). Bone infiltration or erosion didn’t impact LC (*P* = 0.2) or survival (*P* = 0.4) of our patients.

### Toxicities

RT was well tolerated in our cohort without significant adverse events (AE). During the RT courses, almost all patients showed grade 1 toxicity (70%). Only 15% of the patients experienced grade 2 AE (29% with high-dose regimens versus 11% in low-dose group, *P* = 0.6). Grade 2 toxicity rate was lower with IMRT technique in comparison with conventional RT technique (14% versus 33%, *P* = 0.3). Most common AE were erythema, xerostomia, and mucositis. No moderate or severe radiation-related toxicities. There were three radiation breaks (one primary EMP course and 2 secondary EMP).

## Discussion

The hereby presented study reveals one of the largest study collectives of plasmocytomas in the literature and corroborates the role of RT as a curative treatment for EMP. A local control rate of 76% could be achieved which is in accordance with the literatur indicating a control rate between 66 - 100% [4, 6, 7, 9, 13, 20–23]. Importantly, further sub-stratification revealed a significant favour of primary in comparison to secondary EMP regarding LC (93% vs. 61%), CR rate (67% vs. 22%) and 2-year OS rate of 85% vs. 53%. Although belonging to the continuum plasma-cell neoplasms altogether, primary and secondary EMP represent distinct entitities with different behaviour. Primary EMP is a localized disease and curable by local RT, while secondary EMP is a systemic disease.

Only a minority of patients with primary EMP progresses to multiple myeloma (4 -33% after several years) [5, 6, 9, 13, 15, 20, 21, 23] underlining the localized character for primary EMP. No systemic progress to MM could be seen in the patients of this analysis. Interestingly, most transformations occur within the first 5 years with late disseminations being possible [6, 13, 15, 21, 23] . It is tempting to speculate that biologic behaviour of EMP may change during the course of disease with a less aggressive pattern seen in the long-term period.

The present analysis shows a non-significant higher ORR and longer duration of LC with a longer survival for the high-dose group in primary EMP, thus suggesting a clinical benefit of a RT dose above 45 Gy. Defining the adequate radiation dose for EMP has been an issue of debate since several decades leading to the establishment of cutoff-doses.

Various studies postulated cutoff-values between 40 and 49 Gy for improved control or disease-free survival [13, 24–26]. Other analyses often failed to demonstrate a dose-response-relationship or did not provide own insights in dosage [5–7, 27]. In a meta-analysis including 315 patients, a dose-escalation > 45 Gy showed improved disease-free survival [23]. Strojan et al. [21] introduced a differentiated approach demanding 40–50 Gy for macroscopic disease (with no local relapse with > 40), whereas adjuvant RT of 36–40 Gy may be adequate. Taken this data into account, the International Lymphoma Radiation Oncology Group (ILROG) has recently provided comprehensive guidelines on the treatment of MM and EMP recommending a dose between 40 and 50 Gy [17]. One major drawback for dose-definition is the rarity of EMP requiring a patient recrutation of several decades with changes in radiation technique and concepts [21, 25]. Therefore, comparable patient groups concerning dosage could not always be established due to low patient number, biological different RT regimes (split course, hypofractionation) or limited dose ranges/variation [21, 24, 25]. The present analysis is one of the first to introduce a systematic comparison of nearly equivalent RT dose groups and elaborates the differences. A potential bias concerning RT location and subsequent dose adaption did not show a significant impact, probably due to small sample size .

Furthermore, there has been considerable debate on the elective regional lymph nodes irradiation, although a minority of EMP reveals lymph-node involvement in up to one third of cases [9, 28]. Following initial therapy, locoregional recurrences are rare events [6, 9, 13, 20, 28] and may be re-irradiated effectively [9]. The ILROG guidelines renounce an inclusion of lymph node except for bulky disease and tumor adjacent regional lymph-nodes permitting an inclusion within the PTV without a relevant increase in radiation toxicity [17]. Consistently, only 6/33 patients (18%) received elective nodal RT. Novel systemic therapies following RT may improve outcome [29].

To further characterize clinical risk factors, the serological profile before RT was analysed for each patient regarding hemoglobin level, serum calcium, serum protein, immunfixation in urin and serum, β2-microglobulin, LDH. Positive immunfixation, although nonsignificant, may have a worse impact on LC and OS in the present collective, which is debatable in the literature: while some research group identified positive serum protein as a factor heralding progression to MM [27], others negate a significant impact [6, 7].

Within the last years, the chromosomal and molecular analysis of pathogenesis could shed light on the development MM and helped to identify “key players” such as immunoglobulin heavy-chain translocations, cyclin D overexpression or trisomies [30]. Thus, the established international staging system defining risk categories via serum albumin and β2-microglobulin levels may be further stratified on the molecular level c [30, 31].

Our study bears some limitations known to retrospective analysis as the lack of a control group or different treatment group. Furthermore, the number of patients is limited due to the low incidence of the disease which may hamper statistical analysis. In addition by focussing on head and neck plasmocytoma for their predominance in EMP, tumors of other location with possible differentiated biology and response have been excluded.

The optimal radiation dose for EMP warrants further investigations. More clinical and biological data are needed to identify patients who may require neoadjuvant or adjuvant therapies, as well as dose escalation during RT as part of a biologically guided therapeutic strategy. Additionaly, the benefit of a combined modality treatment of RT and proteasome-inhibitor or immunomodulatory agents remain uncertain and may be warranted for patients with incomplete response RT. Currently, a multi-center study is under way to enable a more profound understanding of EMP and its optimal treatment approach.

## Conclusion

Patients with primary EMP manifestations are associated with better outcome compared with secondary EMP. Response to RT might influence the OS of EMP patients. A radiation dose ≤45 Gy confer a comparable CR rate to high-dose regimens and appears to be an effective treatment for controlling EMP progression. Radiation dose-escalation seems to be beneficial for particular subgroups of patients. Further studies with a larger sample size are needed to confirm the results of this analysis.

## Abbreviations

DOLC: Duration of local control
EFS: Event-free survival
EMP: Extramedullary plasmacytoma
LC: Local control
MM: Multiple myeloma
OS: Overall survival
RT: Radiotherapy
